# Dynamic changes in CBC-derived inflammatory indices before and during acute coronary syndrome in patients with rheumatoid arthritis: a within-patient paired study

**DOI:** 10.3389/fimmu.2026.1833991

**Published:** 2026-09-30

**Authors:** Yuwei Wang, Yuying Zhang, Jing Zhang, Weiduo Nie, Xiaolong Wang, Fangcheng Su, Sheng-Guang Li, Yuanyuan Tang, Di Jin

**Affiliations:** 1 Department of Emergency Internal Medicine, Weifang People’s Hospital, Weifang, Shandong, China; 2 Department of Rheumatology, Weifang People’s Hospital, Weifang, China; 3 Department of Rheumatology and Immunology, Peking University International Hospital, Beijing, China; 4 Dongfang Hospital, Beijing University of Chinese Medicine, Beijing, China; 5 Department of Rheumatology, Yidu Central Hospital of Weifang, Weifang, Shandong, China

**Keywords:** acute coronary syndrome, cardiovascular risk, immunothrombosis, inflammation, neutrophil-to-lymphocyte ratio, rheumatoid arthritis, systemic immune-inflammation index, systemic inflammation response index

## Abstract

**Background:**

Rheumatoid arthritis (RA) is associated with excess cardiovascular risk, but within-patient changes in complete blood count (CBC)-derived inflammatory indices at the onset of acute coronary syndrome (ACS) are not well described. We compared pre-event and acute-phase values in the same patients and contextualized pre-event values against a healthy cohort.

**Methods:**

This retrospective, single-center, within-patient paired study reviewed hospital records from July 1, 2018, to December 30, 2025 (approximately 90 months). Among 3,960 hospitalized patients with RA, 89 had clinician-confirmed ACS and qualifying paired CBC measurements. Acute-phase CBCs were obtained within 24 hours after symptom onset, and pre-event CBCs were the most recent routine measurements obtained more than 14 days before the event. The neutrophil-to-lymphocyte ratio (NLR), systemic immune-inflammation index (SII), and systemic inflammation response index (SIRI) were calculated and natural log-transformed. Pre-event values were also compared with a healthy cohort (n = 110), with age- and sex-adjusted analyses used because residual demographic imbalance was present.

**Results:**

Pre-event log(NLR), log(SII), and log(SIRI) were higher in the RA cohort than in healthy controls and remained higher after adjustment for age and sex (all P< 0.001). Within-patient analyses showed increases during ACS, with mean Δlog values of 0.466 for NLR, 0.508 for SII, and 0.381 for SIRI (all Benjamini–Hochberg-adjusted q< 0.001). Neutrophilia increased from 11.2% before the event to 41.6% during ACS (McNemar P< 0.001). Exploratory subgroup estimates were heterogeneous and non-monotonic across several small strata; changes did not differ materially between remission/low and moderate/high RA activity groups.

**Conclusion:**

Among patients with RA who developed ACS, CBC-derived inflammatory indices were higher than in a healthy comparison cohort before the event and increased further within the first 24 hours of ACS. The paired design quantifies change relative to each patient’s own pre-event value. Without a non-RA ACS comparator, however, the study cannot establish RA-specific inflammatory priming or a causal chronic-to-acute mechanism. Prospective studies with serial sampling and clinical outcomes are needed.

## Introduction

Rheumatoid arthritis (RA) is a chronic systemic autoimmune disease in which persistent inflammation accelerates atherosclerosis and contributes substantially to excess cardiovascular morbidity and mortality (1). Among readily available biomarkers, complete blood count (CBC)-derived indices such as the neutrophil-to-lymphocyte ratio (NLR), systemic immune-inflammation index (SII), and systemic inflammation response index (SIRI) provide composite readouts of innate immune activation, lymphocyte suppression, and thrombo-inflammatory burden (2, 3). In RA, these indices are elevated and correlate with inflammatory activity, supporting their value as inexpensive markers of systemic immune dysregulation (2, 3). In acute coronary syndrome (ACS), higher NLR has been associated with greater coronary severity and worse short- and long-term outcomes, suggesting that CBC-derived indices may also reflect the intensity of ischemia-triggered inflammation (4, 5).

However, most RA studies have been cross-sectional, whereas most ACS studies have used a single admission measurement. Comparing acute-phase values with each patient’s own pre-event value quantifies deviation from that individual’s prior state and reduces confounding from stable between-person characteristics. This design does not determine whether RA produces a larger ACS-associated response than occurs in patients without RA. CBC-derived indices are also nonspecific and may be influenced by infection, glucocorticoid exposure, recent trauma or surgery, renal or hematologic disease, and acute treatment. Vascular inflammation, endothelial dysfunction, and immunothrombosis provide plausible biological context, but none was directly measured here. We therefore aimed to describe within-patient changes in NLR, SII, and SIRI from the pre-event period to the first 24 hours of ACS and to provide a contextual comparison with a same-hospital healthy cohort.

## Methods

### Study design and source population

This was a retrospective, single-center, within-patient paired study conducted at a tertiary hospital. We reviewed records from July 1, 2018, to December 30, 2025 (approximately 90 months). Hospital inpatient records identified 3,960 patients with RA during this period. Potential ACS cases were retrieved from recorded discharge diagnoses, and 89 patients met the analytic eligibility criteria described below. The de-identified analytic dataset did not retain the ICD code list or the electronic query syntax used for the initial electronic retrieval; final eligibility and ACS subtype were based on clinician review of the medical record rather than an ICD-only rule.

The study compared complete blood count (CBC)-derived inflammatory indices during two predefined windows within the same patient: a pre-event window and an acute-event window. Each patient served as his or her own control. The objective was descriptive and associational; the design did not include a non-RA ACS group and was not intended to test RA-specific priming or causality.

### Patient eligibility

Patients were eligible if they met all of the following criteria:

1. RA diagnosed according to the 2010 American College of Rheumatology/European League Against Rheumatism (ACR/EULAR) classification criteria (6).
2. A final clinical diagnosis of ACS, classified as ST-segment elevation myocardial infarction (STEMI), non-ST-segment elevation myocardial infarction (NSTEMI), or unstable angina (UA). Diagnosis was based on contemporaneous clinical evaluation, including ischemic symptoms, electrocardiography, serial myocardial-injury biomarkers, and coronary angiography when clinically indicated. Angiography was not an inclusion requirement, and patient-level angiographic variables were not abstracted. The diagnosis and subtype were jointly reviewed by two clinicians.
3. Availability of one stable pre-event CBC and one acute-phase CBC suitable for paired analysis.

The pre-event CBC was the most recent routine CBC obtained during follow-up more than 14 days before the coronary event. The acute-phase CBC was the first CBC obtained within 24 hours after symptom onset; all included acute-phase samples met this window.

A qualifying pair of CBC measurements was required for inclusion. In the final analytic datasets, all 89 cases and all 110 controls had complete values for every variable reported in the tables and analyses; therefore, no imputation was performed and all analyses used the complete available observations. Information on concurrent infection, antibiotic exposure, recent major trauma or surgery, renal function, hematologic disorders, glucocorticoid exposure around ACS, and detailed cardiovascular treatment was not systematically available and could not be used as exclusion criteria or adjustment variables.

### Healthy control cohort

For a supportive baseline comparison, an independent healthy cohort (n = 110) was selected from the Health Examination Center of the same hospital. CBC measurements were performed using the same laboratory system and analytical platform as the RA samples. At blood sampling, the control records contained no documented autoimmune or active inflammatory disorder or overt cardiovascular disease. The control cohort was not individually matched, and age and sex distributions differed between cohorts. Accordingly, unadjusted group summaries were retained for description, and regression models adjusted for age and sex were used for the principal between-group comparison. The control analysis provides context for pre-event values and is separate from the within-patient paired analysis.

### Clinical variables and subgroup definitions

The following variables were extracted from the final analytic dataset for the 89 RA patients with ACS:

- age and sex.
- RA duration.
- RA treatment category at the time of the event, classified as csDMARDs or csDMARDs + bDMARDs.
- metabolic/cardiovascular risk burden, encoded in the source dataset as the number of documented hypertension, diabetes mellitus, and dyslipidemia diagnoses (0–3). This simple count was used only for descriptive subgroup summaries and was not treated as a validated risk score.
- ACS subtype (NSTEMI, STEMI, or UA).
- RA activity-related variables, including erythrocyte sedimentation rate (ESR), C-reactive protein (CRP), Disease Activity Score in 28 joints based on ESR or CRP (DAS28-ESR and DAS28-CRP), Clinical Disease Activity Index (CDAI), and Simplified Disease Activity Index (SDAI), from the most recent recorded rheumatology assessment before ACS. The interval between that assessment and ACS was not retained.

Because the subgroup analyses were exploratory and several subgroups were small, especially the csDMARDs + bDMARDs group and risk category 3, subgroup findings were interpreted descriptively.

### CBC measurements and derived inflammatory indices

All CBC measurements were obtained from the same institutional laboratory, using uniform reference intervals throughout the study period. The reference ranges applied in this study were as follows: white blood cells (WBC): 3.5–9.5× 10^9^/L; neutrophils: 1.8**–**6.3×10^9^/L; lymphocytes: 1.1–3.2×10^9^/L; monocytes: 0.1–0.6×10^9^/L; and platelets: 125–350×10^9^/L.

From absolute cell counts, the following CBC-derived inflammatory indices were calculated: NLR = neutrophils/lymphocytes; SII = platelets × neutrophils/lymphocytes; SIRI = monocytes × neutrophils/lymphocytes;

Because these indices were right-skewed, they were natural log-transformed before inferential analysis. The primary within-patient change was defined as: Δlog(Index)=log(acute)−log(pre-event). A positive Δlog value indicates a higher value during ACS than before the event.

### Statistical analysis

Continuous variables are presented as mean ± SD or median (IQR), and categorical variables as n (%). Pre-event RA values and healthy-control values were log-transformed and compared using Welch’s two-sample t-test. For the principal comparison, linear regression with HC3 standard errors was used for each log-transformed index, adjusted for cohort, age, and sex. For within-patient acute-phase changes (Δlog), time was coded as 0 (pre-event) and 1 (acute); estimates were derived from linear mixed-effects models with patient-specific random intercepts. For the three co-primary outcomes (Δlog NLR, SII, SIRI), P values from paired t-tests were adjusted via Benjamini–Hochberg and reported as q values. Exploratory subgroup analyses (by ACS subtype, RA treatment, metabolic/cardiovascular risk burden, and sex) and RA activity category comparisons (DAS28-ESR ≤3.2 vs. >3.2) were performed; the latter used Welch’s t-test with Benjamini–Hochberg adjustment. Changes in paired abnormal blood counts (neutrophilia, lymphopenia, thrombocytosis) were assessed by McNemar’s test. Correlations between RA activity markers and Δlog indices were evaluated using Pearson coefficients, with nominal P values interpreted by effect size without multiplicity correction. Prespecified sensitivity analyses comprised 1% winsorization of Δlog(SII) and restriction to definite MI cases (STEMI/NSTEMI). All tests were two-sided, with P< 0.05 considered significant. Analyses used Python 3.12.9, SciPy 1.17.1, and statsmodels 0.14.6. No *a priori* sample-size calculation was performed because all patients meeting the analytic eligibility criteria during the study period were included. With 89 paired observations, the study had approximately 80% power at a two-sided α of 0.05 to detect a standardized within-patient mean change (Cohen dz) of 0.30. This sensitivity calculation does not establish adequate power for subgroup comparisons, which remained exploratory.

### Ethics statement

This study was conducted in accordance with the Declaration of Helsinki. The protocol was approved by the Medical Research Ethics Committee of Weifang People’s Hospital, Weifang, China (approval No. KYLL20251029-3; October 29, 2025). Because the study was retrospective and used de-identified clinical data, the committee waived the requirement for written informed consent. All patient data were de-identified before analysis.

## Results

### Cohort derivation and baseline characteristics

During the approximately 90-month study period, hospital records identified 3,960 patients with RA. Of these, 89 had clinician-confirmed ACS and qualifying pre-event and acute-phase CBC measurements and constituted the analytic cohort (**Table 1**). All variables reported for the 89 cases and 110 controls were complete; no imputation was performed.

**Table 1:** Source population and baseline characteristics of patients with rheumatoid arthritis and acute coronary syndrome (n = 89).

Population/characteristic	Value
Study period	July 1, 2018 to December 30, 2025 (approximately 90 months)
Hospitalized RA patients in the source population	3,960
RA patients with ACS and qualifying paired CBCs	89
Age, years	69.8 ± 10.1
Sex	Women: 64 (71.9%); men: 25 (28.1%)
RA duration, months	120 [84–240]
DAS28-ESR activity	Remission/low: 22 (24.7%); moderate/high: 67 (75.3%)
RA treatment	csDMARDs: 87 (97.8%); csDMARDs + bDMARDs: 2 (2.2%)
Metabolic/cardiovascular risk burden	0: 28 (31.5%); 1: 39 (43.8%); 2: 20 (22.5%); 3: 2 (2.2%)
ACS subtype	NSTEMI: 41 (46.1%); STEMI: 35 (39.3%); UA: 13 (14.6%)
Acute-phase CBC window	All samples obtained within 24 hours after symptom onset
Missing observations in reported variables	0; no imputation

Values are mean ± standard deviation, median [interquartile range], or n (%). ACS, acute coronary syndrome; bDMARDs, biologic disease-modifying antirheumatic drugs; CBC, complete blood count; csDMARDs, conventional synthetic disease-modifying antirheumatic drugs; NSTEMI, non-ST-segment elevation myocardial infarction; RA, rheumatoid arthritis; STEMI, ST-segment elevation myocardial infarction; UA, unstable angina. All reported variables were complete for the 89 cases.

The 89 patients were predominantly female (64/89, 71.9%) with a mean age of 69.8 ± 10.1 years. Median RA duration was 120 months [84–240]. Most patients were receiving csDMARDs alone (87/89, 97.8%), whereas 2 patients (2.2%) were receiving csDMARDs + bDMARDs. Metabolic/cardiovascular risk categories were distributed as follows: 28 (31.5%) in category 0, 39 (43.8%) in category 1, 20 (22.5%) in category 2, and 2 (2.2%) in category 3. ACS subtype included 41 NSTEMI cases (46.1%), 35 STEMI cases (39.3%), and 13 UA cases (14.6%) (**Table 1**).

All acute-phase CBC samples were obtained within 24 hours after symptom onset.

### Baseline comparison between pre-event RA samples and healthy controls

The 110 healthy controls had a mean age of 63.1 ± 6.5 years and included 96 women (87.3%), whereas the pre-event RA cohort had a mean age of 69.8 ± 10.1 years and included 64 women (71.9%). Unadjusted mean log(NLR), log(SII), and log(SIRI) were higher in the RA cohort (**Table 2**; **Figure 1A**). After adjustment for age and sex, the between-group coefficients remained 0.445 (95% CI 0.269–0.621; P = 7.70 × 10^-7^) for log(NLR), 0.460 (95% CI 0.274–0.647; P = 1.27 × 10^-6^) for log(SII), and 0.768 (95% CI 0.565–0.970; P = 1.08 × 10^-^¹³) for log(SIRI) (**Table 2**). These comparisons describe the selected cohorts and do not establish that RA caused the between-group differences.

**Table 2:** Pre-event comparison of log-transformed CBC-derived inflammatory indices between patients with rheumatoid arthritis and healthy controls.

Index	Pre-event RA (n = 89), mean ± SD	Healthy controls (n = 110), mean ± SD	Unadjusted difference (95% CI); P	Age/sex-adjusted β (95% CI); P
log (NLR)	1.035 ± 0.653	0.566 ± 0.350	0.469 (0.317–0.621); 1.15 × 10^-8^	0.445 (0.269–0.621); 7.70 × 10^-7^
log (SII)	6.461 ± 0.714	5.992 ± 0.421	0.469 (0.300–0.639); 2.02 × 10^-7^	0.460 (0.274–0.647); 1.27 × 10^-6^
log (SIRI)	0.287 ± 0.721	−0.535 ± 0.497	0.822 (0.644–1.000); 4.07 × 10^-^¹^6^	0.768 (0.565–0.970); 1.08 × 10^-^¹³

Unadjusted differences were estimated using Welch’s t test. Adjusted coefficients were obtained from linear regression with age and sex and HC3 standard errors. The RA cohort was older (69.8 ± 10.1 vs 63.1 ± 6.5 years) and included fewer women (71.9% vs 87.3%) than the control cohort; therefore, the groups are not described as matched. CBC, complete blood count; CI, confidence interval; NLR, neutrophil-to-lymphocyte ratio; RA, rheumatoid arthritis; SD, standard deviation; SII, systemic immune-inflammation index; SIRI, systemic inflammation response index.

**Figure 1:**
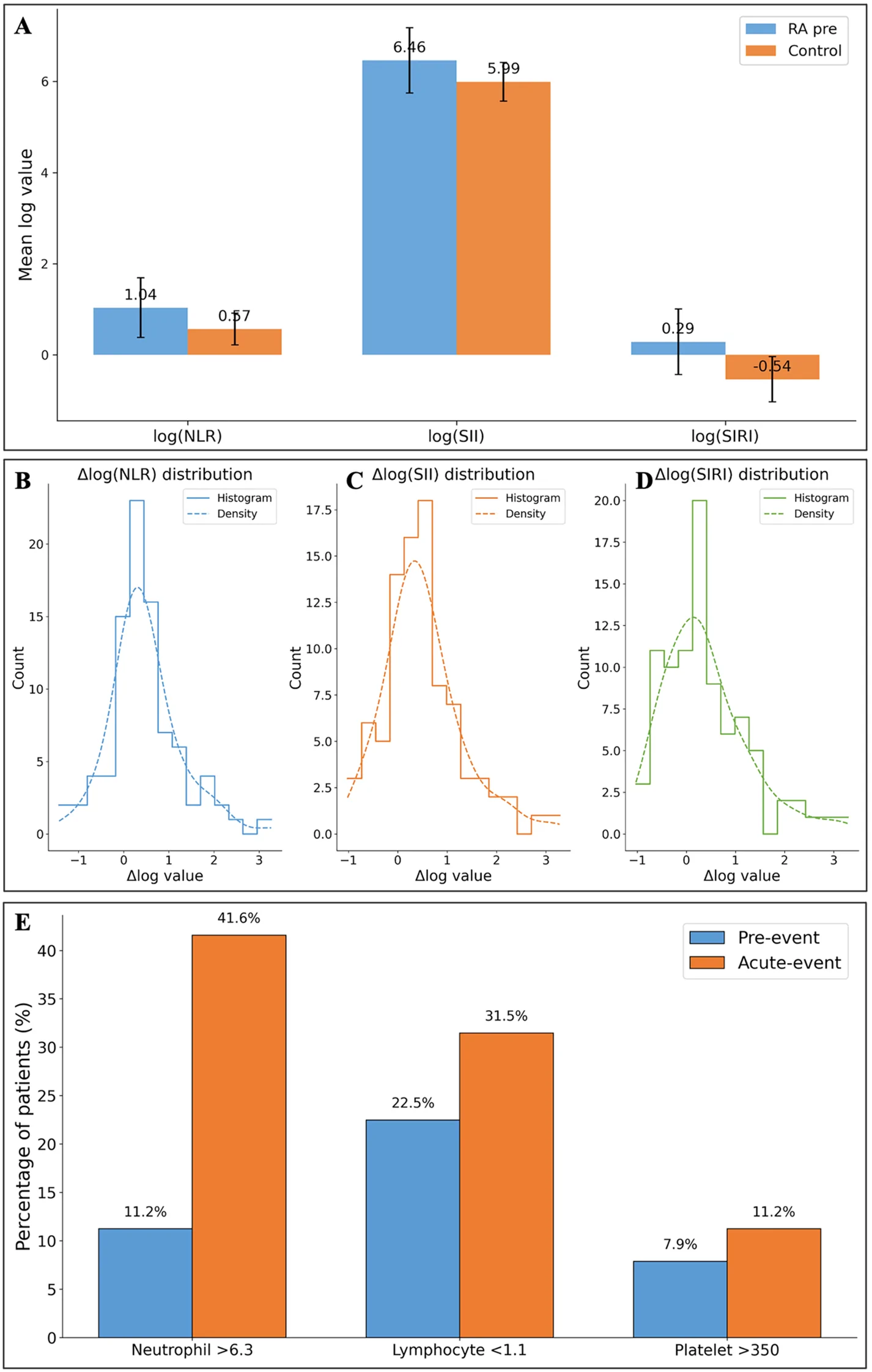
Pre-event inflammatory-index comparison, distributions of within-patient change, and paired shifts in abnormal blood-count proportions. **(A)** Unadjusted mean ± standard deviation of log-transformed neutrophil-to-lymphocyte ratio (NLR), systemic immune-inflammation index (SII), and systemic inflammation response index (SIRI) in patients with rheumatoid arthritis before acute coronary syndrome (RA pre) and healthy controls. Age- and sex-adjusted comparisons are reported in **Table 2**. **(B–D)** Histograms and density curves of Δlog(NLR), Δlog(SII), and Δlog(SIRI), where Δlog = log(acute-phase value) − log(pre-event value). Positive values indicate an increase during acute coronary syndrome. **(E)** Proportions with neutrophilia (>6.3 × 10^9^/L), lymphopenia (<1.1 × 10^9^/L), and thrombocytosis (>350 × 10^9^/L) before and during acute coronary syndrome. Only the paired increase in neutrophilia was statistically significant by McNemar’s test.

### Descriptive paired CBC data before and during ACS

Paired descriptive data showed a clear shift toward neutrophil-dominant inflammation during ACS (**Table 3**). Mean WBC increased from 6.46 ± 1.79 to 8.18 ± 2.94 × 10^9^/L, and mean neutrophil count increased from 4.24 ± 1.63 to 6.13 ± 2.73 × 10^9^/L. Monocyte count remained relatively stable (0.50 ± 0.18 vs 0.49 ± 0.23 × 10^9^/L), whereas lymphocyte count declined slightly (1.54 ± 0.66 vs 1.39 ± 0.63 × 10^9^/L). Platelet count increased modestly from 238.8 ± 81.1 to 250.5 ± 86.1 × 10^9^/L.

**Table 3:** Pre-event and acute-phase CBC counts and derived inflammatory indices in patients with rheumatoid arthritis and acute coronary syndrome.

Variable	Pre-event mean ± SD	Pre-event median [IQR]	Acute-phase mean ± SD	Acute-phase median [IQR]
WBC (×10^9^/L)	6.46 ± 1.79	6.48 [5.21, 7.38]	8.18 ± 2.94	7.72 [6.03, 9.67]
Neutrophils (×10^9^/L)	4.24 ± 1.63	4.09 [3.03, 5.23]	6.13 ± 2.73	5.63 [4.39, 7.28]
Monocytes (×10^9^/L)	0.50 ± 0.18	0.47 [0.37, 0.62]	0.49 ± 0.23	0.44 [0.34, 0.59]
Lymphocytes (×10^9^/L)	1.54 ± 0.66	1.47 [1.12, 1.95]	1.39 ± 0.63	1.30 [0.96, 1.59]
Platelets (×10^9^/L)	238.8 ± 81.1	223 [189, 274]	250.5 ± 86.1	233 [188, 305]
NLR	3.51 ± 2.64	2.68 [1.81, 4.53]	5.92 ± 7.30	4.21 [2.83, 7.09]
SII	819.5 ± 593.5	585.1 [380.2, 1,139.6]	1,512.7 ± 1,973.0	939.7 [654.4, 1,758.5]
SIRI	1.72 ± 1.28	1.21 [0.79, 2.29]	2.55 ± 2.06	2.00 [1.18, 3.27]

Values are shown for 89 paired observations. CBC, complete blood count; IQR, interquartile range; NLR, neutrophil-to-lymphocyte ratio; SD, standard deviation; SII, systemic immune-inflammation index; SIRI, systemic inflammation response index; WBC, white blood cell count.

Consistent with these raw changes, derived inflammatory indices increased during ACS. Mean NLR rose from 3.51 ± 2.64 to 5.92 ± 7.30, mean SII from 819.5 ± 593.5 to 1,512.7 ± 1,973.0, and mean SIRI from 1.72 ± 1.28 to 2.55 ± 2.06.

### Primary paired analyses of log-transformed inflammatory indices

Within-patient paired analyses showed increases in all three co-primary outcomes (**Table 4**; **Figures 2A–C**). Mean Δlog(NLR) was 0.466 (paired t-test P = 2.07 × 10^-7^; q = 3.10 × 10^-7^; Wilcoxon P = 1.15 × 10^-7^). Mean Δlog(SII) was 0.508 (paired t-test P = 3.65 × 10^-8^; q = 1.09 × 10^-7^; Wilcoxon P = 6.33 × 10^-8^), and mean Δlog(SIRI) was 0.381 (paired t-test P = 7.33 × 10^-5^; q = 7.33 × 10^-5^; Wilcoxon P = 5.80 × 10^-4^). The corresponding standardized within-patient mean changes (Cohen dz) were 0.60, 0.64, and 0.44, respectively.

**Table 4:** Primary within-patient analyses of log-transformed inflammatory indices in patients with rheumatoid arthritis and acute coronary syndrome.

Outcome	Pre-event mean ± SD	Acute-phase mean ± SD	Mean Δlog	Paired t P; BH q	Wilcoxon P	LMM β (95% CI); P
log (NLR)	1.035 ± 0.653	1.502 ± 0.667	0.466	2.07 × 10^-7^; 3.10 × 10^-7^	1.15 × 10^-7^	0.466 (0.304–0.628); 1.74 × 10^-8^
log (SII)	6.461 ± 0.714	6.969 ± 0.774	0.508	3.65 × 10^-8^; 1.09 × 10^-7^	6.33 × 10^-8^	0.508 (0.343–0.673); 1.57 × 10^-9^
log (SIRI)	0.287 ± 0.721	0.667 ± 0.747	0.381	7.33 × 10^-5^; 7.33 × 10^-5^	5.80 × 10^-4^	0.381 (0.202–0.560); 3.15 × 10^-5^

All analyses used 89 paired observations. q values are Benjamini–Hochberg adjustments of the three paired t-test P values. CI, confidence interval; LMM, linear mixed-effects model; NLR, neutrophil-to-lymphocyte ratio; SD, standard deviation; SII, systemic immune-inflammation index; SIRI, systemic inflammation response index.

**Figure 2:**
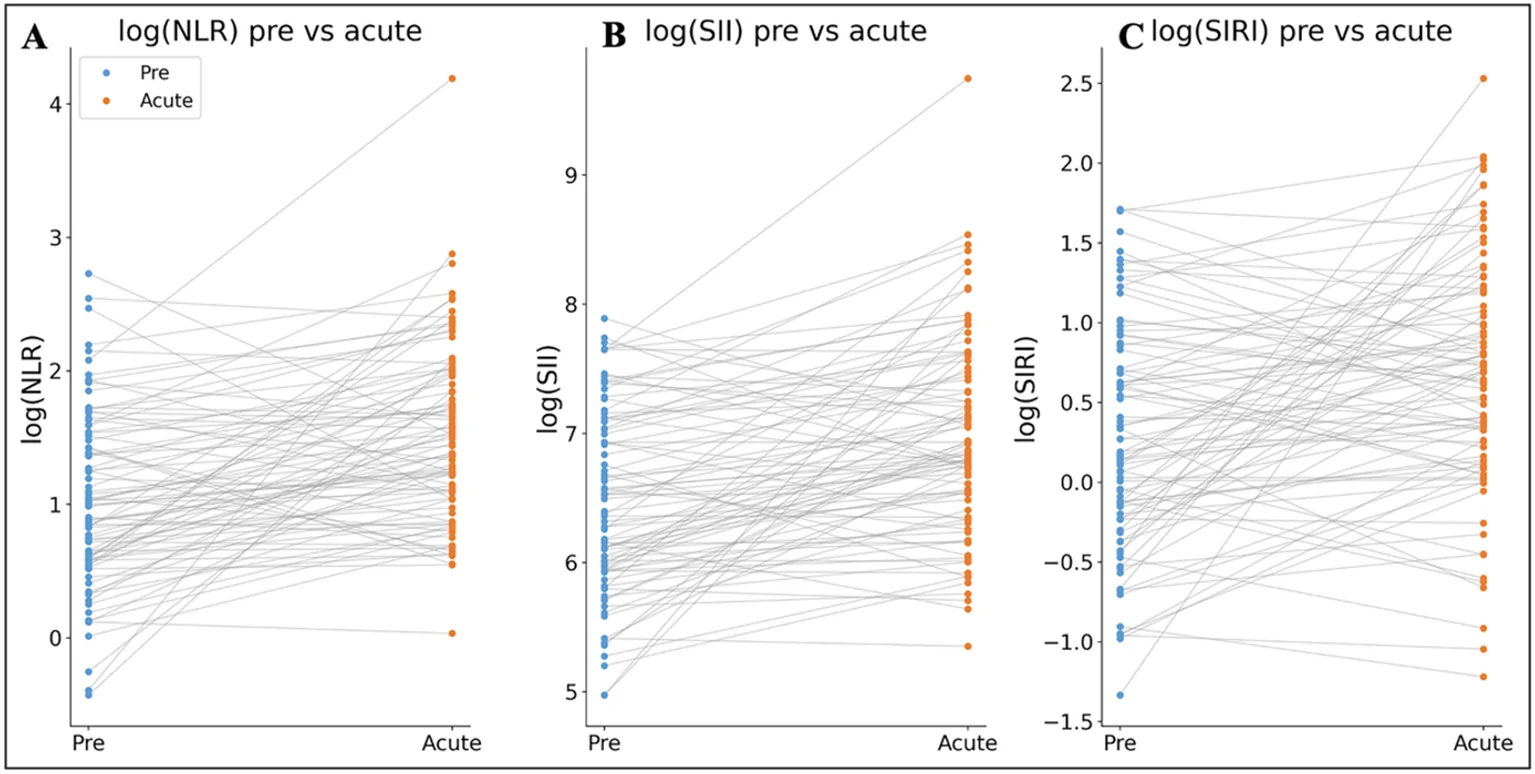
Within-patient paired changes in complete blood count-derived inflammatory indices before and during acute coronary syndrome in patients with rheumatoid arthritis. **(A–C)** Individual changes in log(NLR), log(SII), and log(SIRI) from the pre-event to the acute phase. Each gray line represents one patient; blue points indicate pre-event values and orange points indicate acute-phase values. NLR, neutrophil-to-lymphocyte ratio; SII, systemic immune-inflammation index; SIRI, systemic inflammation response index.

Linear mixed-effects models yielded the same direction of change: β = 0.466 (95% CI 0.304–0.628) for log(NLR), β = 0.508 (95% CI 0.343–0.673) for log(SII), and β = 0.381 (95% CI 0.202–0.560) for log(SIRI) (**Table 4**). These estimates document early within-patient hematologic changes during ACS; they do not distinguish a general ACS response from an RA-specific response.

The distributions of Δlog(NLR), Δlog(SII), and Δlog(SIRI) were predominantly shifted toward positive values, indicating that most patients experienced an increase in inflammatory indices during ACS (**Figures 1B–D**).

### Exploratory subgroup analyses

Mean Δlog estimates varied across ACS subtype, treatment category, metabolic/cardiovascular risk burden, and sex (**Table 5**; **Figures 3A–C**). STEMI had numerically higher mean estimates than NSTEMI and UA. Estimates across metabolic/cardiovascular risk categories were non-monotonic, and the csDMARDs + bDMARDs group and risk category 3 each contained only two patients. No formal interaction tests were performed; these subgroup results are descriptive.

**Table 5:** Exploratory subgroup summaries of within-patient Δlog inflammatory indices.

Subgroup domain	Subgroup	n	Δlog(NLR), mean ± SE	Δlog(SII), mean ± SE	Δlog(SIRI), mean ± SE
ACS subtype	NSTEMI	41	0.357 ± 0.139	0.443 ± 0.148	0.291 ± 0.143
	STEMI	35	0.632 ± 0.112	0.648 ± 0.102	0.488 ± 0.145
	UA	13	0.365 ± 0.188	0.338 ± 0.195	0.375 ± 0.201
RA treatment	csDMARDs	87	0.470 ± 0.085	0.514 ± 0.086	0.389 ± 0.093
	csDMARDs + bDMARDs	2	0.320 ± 0.236	0.252 ± 0.262	0.039 ± 0.407
Risk burden	0	28	0.637 ± 0.171	0.740 ± 0.165	0.468 ± 0.187
	1	39	0.279 ± 0.109	0.283 ± 0.111	0.220 ± 0.119
	2	20	0.541 ± 0.171	0.603 ± 0.183	0.528 ± 0.201
	3	2	0.977 ± 0.188	0.718 ± 0.101	0.816 ± 0.597
Sex	Male	25	0.210 ± 0.135	0.224 ± 0.152	0.087 ± 0.136
	Female	64	0.566 ± 0.100	0.619 ± 0.098	0.496 ± 0.113
DAS28-ESR activity	Remission/low (≤3.2)	22	0.508 ± 0.092	0.564 ± 0.095	0.415 ± 0.147
	Moderate/high (>3.2)	67	0.453 ± 0.106	0.490 ± 0.108	0.370 ± 0.112

Values are mean ± standard error. For the DAS28-ESR activity comparison, Welch’s t-test P values and Benjamini–Hochberg q values for NLR, SII, and SIRI were 0.694/0.808, 0.604/0.808, and 0.808/0.808, respectively. No formal interaction tests were performed. ACS, acute coronary syndrome; bDMARDs, biologic disease-modifying antirheumatic drugs; csDMARDs, conventional synthetic disease-modifying antirheumatic drugs; DAS28-ESR, Disease Activity Score in 28 joints based on erythrocyte sedimentation rate; NSTEMI, non-ST-segment elevation myocardial infarction; SE, standard error; STEMI, ST-segment elevation myocardial infarction; UA, unstable angina. The csDMARDs + bDMARDs and risk-category 3 groups each contained two patients.

**Figure 3:**
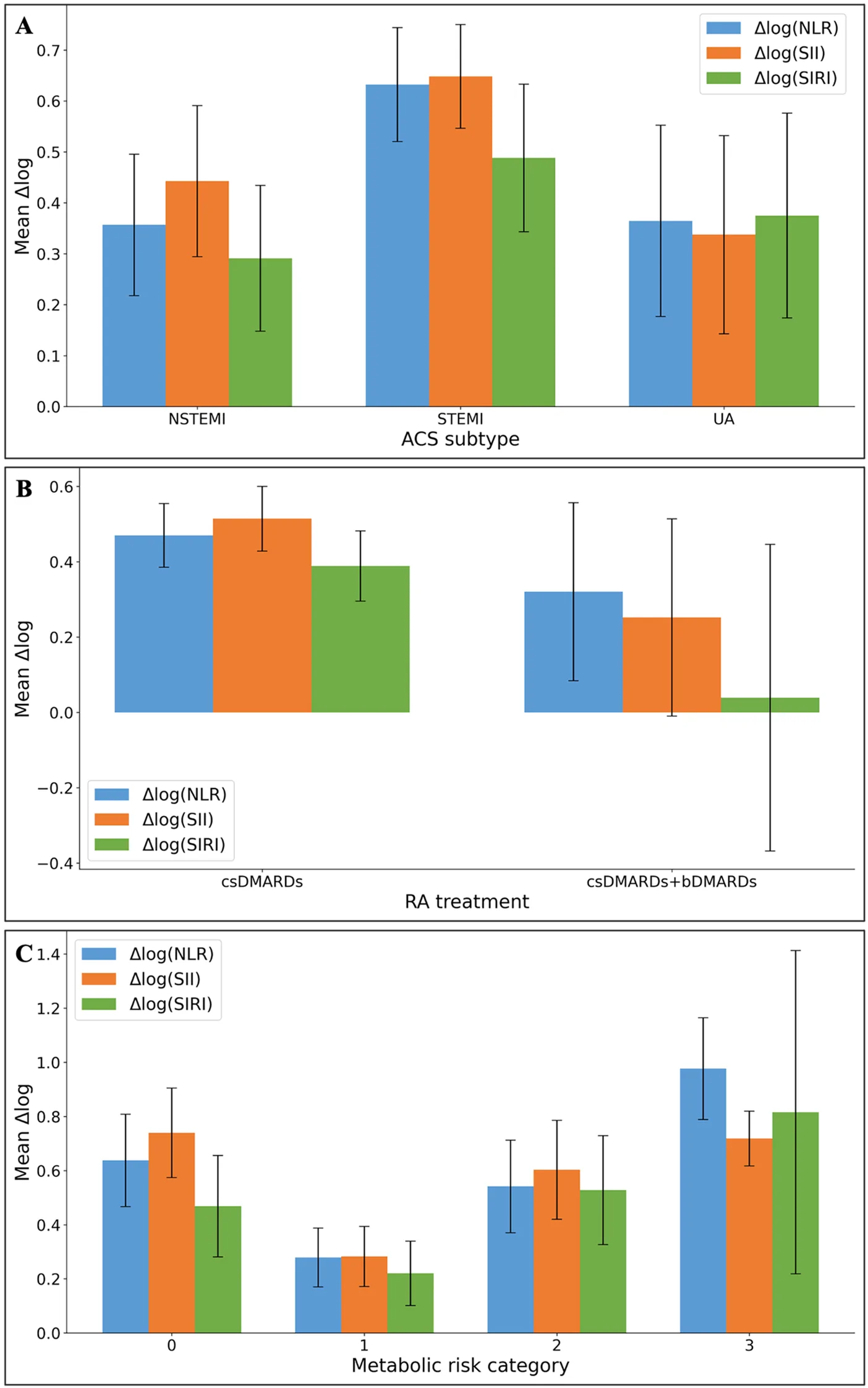
Exploratory subgroup summaries of within-patient changes in complete blood count-derived inflammatory indices. **(A)** Mean Δlog(NLR), Δlog(SII), and Δlog(SIRI) by acute coronary syndrome subtype: ST-segment elevation myocardial infarction (STEMI), non-ST-segment elevation myocardial infarction (NSTEMI), and unstable angina (UA). **(B)** Mean Δlog values according to rheumatoid arthritis treatment regimen: conventional synthetic disease-modifying antirheumatic drugs (csDMARDs) alone or csDMARDs plus biologic DMARDs (bDMARDs). **(C)** Mean Δlog values according to metabolic/cardiovascular risk burden (0–3). Estimates were non-monotonic; the csDMARDs + bDMARDs group and risk category 3 each included two patients. Δlog = log(acute-phase value) − log(pre-event value). Error bars indicate standard error. All subgroup analyses are descriptive; no formal interaction tests were performed.

In the exploratory RA-activity comparison, 22 patients were in remission or had low activity (DAS28-ESR ≤3.2) and 67 had moderate or high activity. Mean Δlog values were similar between these groups for NLR (0.508 vs 0.453; P = 0.694; q = 0.808), SII (0.564 vs 0.490; P = 0.604; q = 0.808), and SIRI (0.415 vs 0.370; P = 0.808; q = 0.808) (**Table 5**). Thus, the available data did not show greater acute change among patients with higher recorded RA activity.

### Transitions in abnormal blood count categories

Paired transition analyses showed the most pronounced abnormality shift for neutrophils (**Table 6**; **Figure 1E**). The proportion of patients with neutrophilia (>6.3 × 10^9^/L) increased from 10/89 (11.2%) before the event to 37/89 (41.6%) during ACS. A total of 29 patients transitioned from normal to abnormal, whereas only 2 patients transitioned from abnormal to normal (McNemar P< 0.001) (**Table 6**).

**Table 6:** Paired transitions in abnormal blood-count categories from the pre-event to acute phase.

Marker	Pre-event abnormal, n (%)	Acute-phase abnormal, n (%)	Normal → abnormal, n (%)	Abnormal → normal, n (%)	Exact McNemar P
Neutrophils >6.3 × 10^9^/L	10 (11.2%)	37 (41.6%)	29 (32.6%)	2 (2.2%)	<0.001
Lymphocytes<1.1 × 10^9^/L	20 (22.5%)	28 (31.5%)	17 (19.1%)	9 (10.1%)	0.169
Platelets >350 × 10^9^/L	7 (7.9%)	10 (11.2%)	7 (7.9%)	4 (4.5%)	0.549

Percentages use the full paired cohort (n = 89) as denominator. Exact McNemar tests compare discordant pairs. Only the increase in neutrophilia was statistically significant. L, liter.

For lymphopenia (<1.1 × 10^9^/L), the proportion increased from 20/89 (22.5%) to 28/89 (31.5%). The number of normal-to-abnormal transitions was 17, versus 9 abnormal-to-normal transitions (McNemar P = 0.169) (**Table 6**).

For thrombocytosis (>350 × 10^9^/L), the proportion increased from 7/89 (7.9%) to 10/89 (11.2%), with 7 normal-to-abnormal and 4 abnormal-to-normal transitions (McNemar P = 0.549). These results indicate that the acute event was characterized primarily by a marked shift toward neutrophil elevation (**Figure 1E**).

### Correlations with RA activity markers

Correlations between RA activity markers and Δlog indices were small (**Table 7**). ESR and CRP showed weak positive correlations with Δlog(NLR) and Δlog(SII), whereas correlations involving composite activity scores were close to zero. All nominal P values are shown in **Table 7**. These exploratory correlations describe association and should not be interpreted as predictive performance.

**Table 7:** Exploratory correlations between rheumatoid arthritis activity markers and within-patient Δlog indices.

RA activity marker	n	Δlog (NLR), r (P)	Δlog (SII), r (P)	Δlog (SIRI), r (P)
ESR	89	0.161 (0.131)	0.191 (0.073)	0.123 (0.250)
CRP	89	0.178 (0.096)	0.191 (0.074)	0.126 (0.240)
DAS28-ESR	89	0.030 (0.781)	0.027 (0.802)	−0.025 (0.816)
DAS28-CRP	89	0.069 (0.523)	0.067 (0.531)	0.001 (0.994)
CDAI	89	−0.023 (0.831)	−0.040 (0.711)	−0.083 (0.438)
SDAI	89	0.033 (0.757)	0.023 (0.830)	−0.032 (0.763)

Values are Pearson r with nominal two-sided P values in parentheses; all 89 observations were complete. The 18 correlations were exploratory and were not adjusted for multiplicity. CDAI, Clinical Disease Activity Index; CRP, C-reactive protein; DAS28, Disease Activity Score in 28 joints; ESR, erythrocyte sedimentation rate; NLR, neutrophil-to-lymphocyte ratio; SDAI, Simplified Disease Activity Index; SII, systemic immune-inflammation index; SIRI, systemic inflammation response index.

### Sensitivity analyses

Sensitivity analyses supported the robustness of the primary findings (**Table 8**). After 1% winsorization of Δlog(SII), the mean Δlog(SII) remained 0.506, and the paired comparison remained statistically significant (P< 0.001) (**Table 8**), indicating that the main SII result was not driven by extreme values.

**Table 8:** Sensitivity analyses of within-patient changes in log-transformed inflammatory indices.

Sensitivity analysis	Outcome	n	Mean Δlog (95% CI)	P
1st/99th percentile winsorization	SII	89	0.506 (0.342–0.669)	2.41 × 10^-8^
MI only (excluding UA)	NLR	76	0.484 (0.301–0.666)	1.25 × 10^-6^
MI only (excluding UA)	SII	76	0.537 (0.352–0.722)	1.56 × 10^-7^
MI only (excluding UA)	SIRI	76	0.382 (0.179–0.585)	3.52 × 10^-4^

Confidence intervals and P values were calculated using one-sample t inference on Δlog values. MI, myocardial infarction; NLR, neutrophil-to-lymphocyte ratio; SII, systemic immune-inflammation index; SIRI, systemic inflammation response index; UA, unstable angina.

When the analysis was restricted to definite myocardial infarction cases only (STEMI + NSTEMI, n = 76) and UA was excluded, the mean Δlog values remained highly consistent with the main analysis: 0.484 for NLR, 0.537 for SII, and 0.382 for SIRI (**Table 8**). These findings indicate that the principal results were not dependent on inclusion of UA.

Because all acute-phase CBC samples were obtained within 24 hours of symptom onset, no additional sensitivity analysis based on narrower or broader acute sampling windows was performed.

## Discussion

### Principal findings

This study provides two descriptive observations. First, pre-event log-transformed NLR, SII, and SIRI were higher in the selected RA cohort than in the healthy cohort, including after age and sex adjustment. Second, all three indices increased within the same patients during the first 24 hours of ACS. The paired design is valuable because it measures deviation from each patient’s own prior value and reduces confounding by stable between-person characteristics. It does not, however, show that chronic RA inflammation primes a larger acute response than would occur in patients without RA, because a non-RA ACS comparator was not included.

### Chronic inflammatory background in RA and cardiovascular vulnerability

The higher pre-event log(NLR), log(SII), and log(SIRI) values in the selected RA cohort than in healthy controls are consistent with the broader inflammatory and cardiovascular context of RA, although the comparison remains observational. RA is associated with accelerated atherosclerosis, endothelial dysfunction, oxidative stress, and interactions between inflammation and conventional cardiovascular risk factors (1, 7, 8). Current EULAR recommendations for cardiovascular risk management across rheumatic and musculoskeletal diseases emphasize systematic assessment and management of conventional risk factors (9), while updated EULAR RA-management recommendations require attention to cardiovascular and thromboembolic safety when selecting therapy (10). The adjusted baseline comparison is compatible with, but does not mechanistically test, this framework.

### Acute inflammatory amplification during ACS

All three log-transformed indices increased within the first 24 hours of ACS. This narrow sampling window identifies early-phase CBC changes. However, without serial sampling or comprehensive information on infection, acute glucocorticoid exposure, procedures, and other time-varying factors, the changes cannot be attributed specifically to immunothrombotic activation. In non-RA populations, NLR and SII have been associated with ACS severity and adverse outcomes (4, 11–13). The present findings add within-patient temporal information in RA but do not establish prognostic performance.

### Neutrophils as the biological bridge

Neutrophils provide one biologically plausible link between RA-associated inflammation and coronary instability. They can influence endothelial cells, monocytes, platelets, and adaptive immune cells and contribute to both chronic inflammation and acute tissue injury (14). Because NLR, SII, and SIRI all incorporate neutrophil counts, these indices are sensitive to neutrophil-predominant hematologic change. Consistent with that property, neutrophilia showed the clearest normal-to-abnormal transition during ACS. These observations do not establish vascular inflammation or vasculitis as the mechanism in this cohort.

### NETosis, immunothrombosis, and plaque instability

Neutrophil extracellular trap (NET) formation provides another biologically plausible hypothesis. NETs have been implicated in RA immune activation, atherosclerotic plaque biology, and thrombosis (15, 16). The observed CBC pattern is compatible with, but cannot demonstrate, these pathways. NET-related markers, vascular imaging of inflammation, and evidence of vasculitis were not measured in this study.

### Interpreting subgroup heterogeneity

The subgroup estimates should be interpreted cautiously. STEMI showed numerically higher mean Δlog values than NSTEMI and UA, but no formal interaction tests were performed. The estimates across metabolic/cardiovascular risk categories were non-monotonic, and two subgroups contained only two patients. Apparent differences by sex or treatment are therefore unstable descriptive signals, not evidence of effect modification or mechanism.

### Why correlations with RA activity were weak

The weak correlations between Δlog indices and ESR, CRP, DAS28, CDAI, or SDAI, together with the similar mean changes in remission/low versus moderate/high DAS28-ESR groups, do not support greater acute amplification among patients with higher recorded RA activity. Interpretation remains limited because activity measures came from the most recent pre-event assessment and the interval to ACS was not retained. These data therefore cannot determine whether cumulative or time-varying RA activity modifies the ACS-associated response.

### Clinical implications

CBC-derived inflammatory indices are inexpensive and widely available. In this cohort, they served as research measures of within-patient hematologic inflammatory change. They should not replace troponin, cardiac imaging, or established risk assessment, and the present study does not validate them for clinical stratification. Serial prospective studies are required to determine whether their trajectories add prognostic information beyond routine ACS care.

### Therapeutic implications

Whether antirheumatic therapy modifies ACS-associated CBC changes remains unresolved. Although several therapies have been associated with cardiovascular outcomes in RA (17–22), the present treatment comparison included only two patients receiving csDMARDs + bDMARDs and cannot support treatment-effect inference. This question requires adequately powered longitudinal cohorts with treatment timing and cardiovascular outcomes.

### Strengths, limitations, and future directions

This study’s principal strength is the within-patient paired design, which reduces confounding from stable between-person differences. The source population covered approximately 90 months, and all acute CBCs were obtained within 24 hours after symptom onset. The analysis also included a same-hospital healthy comparison, paired abnormal-count transitions, and prespecified sensitivity analyses.

The study was retrospective, single-center, and modest in size, particularly for subgroup analyses. A non-RA ACS comparator was unavailable, so RA-specific priming cannot be separated from the inflammatory response to ACS in general. The healthy cohort differed in age and sex; regression adjustment reduced but cannot eliminate selection bias or residual confounding. Infection, peri-event glucocorticoids, recent surgery or trauma, renal function, hematologic conditions, detailed ACS treatment, and the precise pre-event interval were not systematically available. Infection, physiologic stress, and glucocorticoids could raise neutrophils and lower lymphocytes, potentially exaggerating acute increases; the direction of bias from other unmeasured factors is uncertain. Initial electronic retrieval details and excluded potential cases could not be reconstructed from the de-identified file. We also lacked serial post-ACS measurements and systematically ascertained outcomes such as heart failure, cardiogenic shock, reinfarction, and time-to-death. Future multicenter prospective studies should include non-RA ACS comparators, serial CBC-derived indices, detailed time-varying covariates, mechanistic markers, and adjudicated outcomes.

## Conclusion

In conclusion, patients with RA who developed ACS had higher pre-event CBC-derived inflammatory indices than a same-hospital healthy comparison cohort and showed further within-patient increases during the first 24 hours of ACS. These findings quantify early hematologic inflammatory change relative to each patient’s own prior value. They do not establish RA-specific priming, a causal chronic-to-acute transition, or clinical prognostic utility. Prospective studies with non-RA ACS comparators, serial measurements, and adjudicated outcomes are needed before these indices can be used for risk stratification.

## Data Availability

The original contributions presented in the study are included in the article/supplementary material. Further inquiries can be directed to the corresponding authors.
